# Cellular and Subcellular Immunohistochemical Localization and Quantification of Cadmium Ions in Wheat (*Triticum aestivum*)

**DOI:** 10.1371/journal.pone.0123779

**Published:** 2015-05-05

**Authors:** Wei Gao, Tiegui Nan, Guiyu Tan, Hongwei Zhao, Weiming Tan, Fanyun Meng, Zhaohu Li, Qing X. Li, Baomin Wang

**Affiliations:** 1 College of Agronomy and Biotechnology, China Agricultural University, Beijing, China; 2 College of Resources and Environmental Sciences, Henan Agricultural University, Zhengzhou, China; 3 Department of Molecular Biosciences and Bioengineering, University of Hawaii at Manoa, Honolulu, Hawaii, United States of America; 4 Institute of Natural Medicine and Chinese Medicine Resources, Beijing Normal University, Beijing, China; Sabanci University, TURKEY

## Abstract

The distribution of metallic ions in plant tissues is associated with their toxicity and is important for understanding mechanisms of toxicity tolerance. A quantitative histochemical method can help advance knowledge of cellular and subcellular localization and distribution of heavy metals in plant tissues. An immunohistochemical (IHC) imaging method for cadmium ions (Cd^2+^) was developed for the first time for the wheat *Triticum aestivum* grown in Cd^2+^-fortified soils. Also, 1-(4-Isothiocyanobenzyl)-ethylenediamine-N,N,N,N-tetraacetic acid (ITCB-EDTA) was used to chelate the mobile Cd^2+^. The ITCB-EDTA/Cd^2+^ complex was fixed with proteins *in situ* via the isothiocyano group. A new Cd^2+^-EDTA specific monoclonal antibody, 4F3B6D9A1, was used to locate the Cd^2+^-EDTA protein complex. After staining, the fluorescence intensities of sections of Cd^2+^-positive roots were compared with those of Cd^2+^-negative roots under a laser confocal scanning microscope, and the location of colloidal gold particles was determined with a transmission electron microscope. The results enable quantification of the Cd^2+^ content in plant tissues and illustrate Cd^2+^ translocation and cellular and subcellular responses of *T*. *aestivum* to Cd^2+^ stress. Compared to the conventional metal-S coprecipitation histochemical method, this new IHC method is quantitative, more specific and has less background interference. The subcellular location of Cd^2+^ was also confirmed with energy-dispersive X-ray microanalysis. The IHC method is suitable for locating and quantifying Cd^2+^ in plant tissues and can be extended to other heavy metallic ions.

## Introduction

Cadmium ion (Cd^2+^) is one of the most toxic elements to animals and humans. Cd^2+^is commonly used in batteries, pigments, electroplating and coating materials, plastics, alloys and phosphors in color television tubes [1]. The sources of Cd^2+^ in agricultural soils include sewage sludge, herbicides and fertilizers. Crops grown in soil contaminated with heavy metals are an important route of entry for toxic heavy metals into the human food chain [2, 3].

As a major crop worldwide, wheat (*Triticum aestivum*) is considered a model plant in toxicological tests and can accumulate much higher amounts of Cd^2+^ than other crops [4]. Metal distribution in plant tissues and cells is important for understanding the mechanisms of tolerance and phytoremediation. Subcellular distribution and chemical forms of chromium, for example, are closely associated with its toxicity [5]. As the first barrier to Cd^2+^ toxicity, most plant species accumulate Cd^2+^ in the roots to restrict its transport to shoots [6]. In wheat, over 80% of the total Cd^2+^ load has been found in the roots [7].

Compartmentalization and chemical forms of heavy metals are correlated with their bio-toxicity. Exact subcellular localization of heavy metals will help reflect their role in plant’s physiology and ecology, demonstrate their uptake and transport process, and clarify the detoxification in plants [8]. Understanding these mechanisms is necessary for reducing heavy metal concentration in crops [9].

Microanalytical methods are available for visual in situ analyses of heavy metals. Autometallography [2,3,10] has been widely used to determine the localization of Cd^2+^. Its staining relies on the formation of metal sulfide precipitates in tissue during fixation by exposure of metallic ions to Na_2_S followed by a silver-developing process where the free metallic ions are located. However, the metal-S coprecipitation reaction is not specific, so positive results are obtained not only with Cd^2+^ but also with Fe^2+^, Zn^2+^ or Cu^2+^ [11,12]. In addition, metal-S coprecipitation requires high concentrations of sulfide and high pH to fix the target metals, which can cause high background interference. Autoradiography has also been used to locate Cd^2+^ [13,14]. However, this method has low spatial resolution due to radiation scattering, complex operation, and radiation effects. When the radiation reaches a certain level, it will change the normal physiological state and cause radiation damage in the plant. Therefore, radionuclide imaging techniques can provide little or no information regarding endogenous transition metal levels and their distribution under normal physiological conditions [15]. Proton-induced X-ray emission (PIXE) analysis [16,17] cannot be used at low metal concentrations and is not adequate for the subcellular localization of metallic ions because of limited spatial resolution. Nonspecificity of the fluorescent dyes for the target metals is a major obstacle for the detection of metallic ions in plant tissues with fluorescence spectroscopy. For example, Leadmium^TM^ Green AM dye is sensitive not only to Cd but also to Pb, and the fluorescence intensities of the dyes in response to metal concentrations are not linear [18,19].

Another method to locate heavy metals is to determine the content in isolated cell compartments [14]. The disadvantages of this method are that it cannot be used for *in situ* visual analyses, and cell components cannot be completely separated, which would influence accuracy and precision of the results. These methods have a major problem of non-specificity, so the results have to be confirmed by energy-disperse X-ray microanalysis (EDXMA). EDXMA, which is based on the emission of X-rays, offers physical measurements of elemental species and their contents [14,15,20].

Compared to these localization methods, the distinct advantage of the immunohistochemical (IHC) method is its specific recognition of target analytes by monoclonal antibodies (mAbs). The technique has been used in studies of macromolecules, such as cytochromes [21], pectins [22], oxidized proteins [23], sulfurtransferase [24],glycine-rich proteins [25] and small bioactive molecules, such as indole-3-acetic acid [26,27], gibberellic acid 3 [28] and abscisic acid [29]. The accurate localization plays a great role in illustrating the physiological function of these compounds in the plant. To our knowledge, the IHC method has not been used to determine the localization of heavy metal ions.

The present study used the derivative of ethylenediamine-N,N,N,N-tetraacetic acid (EDTA), 1-(4-Isothiocyanobenzyl)-EDTA (ITCB-EDTA) to immobilize Cd^2+^ onto proteins, to achieve *in situ* fixation of Cd^2+^ in plant tissue. The formed Cd^2+^-EDTA protein complex can be recognized by anti-Cd^2+^-EDTA mAb4F_3_B_6_D_9_A_1_ [30], which is specific and sensitive to Cd^2+^-EDTA. On this basis, We developed a selective and reliable IHC method of Cd^2+^ detection in plant, compare this method with the conventional metal-S coprecipitation method confirmed with EDXMA, and finally explore the tissue and cellular localization of Cd^2+^ in wheat roots grown in Cd^2+^-fortified soil. In the present study, wheat plants cultivated in soil without Cd^2+^ are called Cd^2+^-negative, while those cultivated in soil fortified with Cd^2+^ are called Cd^2+^-positive.

## Materials and Methods

### Reagents and buffers

ITCB-EDTA, cadmium chloride (CdCl_2_), bovine serum albumin (BSA), anti-mouse IgG-gold antibody, and LR white resin were purchased from Sigma-Aldrich (St. Louis, MO). The FITC-conjugated goat anti-mouse secondary antibody was from Zhongshan Golden bridge Biotechnology Co., LTD (Beijing, China). All other chemicals were of analytical grade and obtained from Beijing Chemical Reagents Co. (Beijing).

Buffers and solutions used include a formaldehyde-acetic acid-alcohol (FAA) solution (50 mL of formaldehyde 40%+ 50 mL of acetic acid + 900 mL of 50% alcohol); phosphate-buffered saline (PBS) (0.1 M phosphate buffer containing 0.9% sodium chloride, pH 7.5); PBS with 0.1% (v/v) Tween-20 (PBST); and PBST containing 0.5% (w/v) gelatin (PBSTG).

### Plant culture and exposure to cadmium

The soil was a mixture of purchased nutrient soil and vermiculite (1:1, v/v), air dried in sunlight and passed through a 5-mm mesh polyethylene sieve to remove large stones and grass debris. Seven treatments (one control and 6 experimental) were tested in triplicate in a randomized block design. The control was not supplemented with Cd^2+^; and the others were fortified with different concentrations of CdCl_2_ solutions followed by a 7-day incubation at ambient temperature as described in Table 1. Wheat seeds were sown and grown in a greenhouse under natural light conditions with day/night temperatures of 25/15°C. Pots (14 cm x 14 cm) were watered once every three days at 250 mL of tap water per time per pot, which was sufficient to saturate the soil to field water capacity without leaching. No additional fertilizers were applied to the soil during the experiment.

**Table 1:** Accumulation and translocation of cadmium ions (Cd^2+^) in the roots and leaves of wheat plants exposed to Cd^2+^ at different concentrations.

Treatments	Concentrations of Cd^2+^ added to the soil (μg g^-1^ DW)^a^	Concentrations of Cd^2+^ detected in roots (μg g^-1^ DW)		Concentrations of Cd^2+^ detected in leaves (μg g^-1^ DW)		Translocation factors^b^ from roots to leaves
		icELISA	GFAAS	icELISA	GFAAS	
Control	0.00	2.96±2.00	2.67±0.10	2.95±0.10	2.18±0.00	0.998
C1	1.00	11.9±5.90	9.21±2.30	4.10±0.00	3.75±0.12	0.343
C2	5.00	78.5±20.0	69.2±11.2	10.1±1.70	12.5±3.20	0.129
C3	25.0	173±21.8	161±22.7	29.2±3.1	27.1±1.9	0.169
C4	50.0	204±20.9	190±18.8	30.9±2.7	32.1±2.1	0.151
C5	100	257±6.1	261±9.21	53.5±2.2	48.7±5.1	0.208
C6	200	301±9.5	303±16.2	44.4±1.8	45.2±6.1	0.148

^a^ The plants grew in soil fortified with Cd^2+^ at different concentrations (means ± SD, n = 5).

^b^ Translocation factors were determined according to the results obtained by icELISA.

### Enzyme linked immunosorbent assay (ELISA) analysis of Cd^2+^

The collected wheat plant samples were carefully washed with deionized water, and the roots and leaves of the wheat plants were separated with plastic scissors; the plant samples were dried at 80°C to a constant weight. The Cd^2+^ content in all samples was then determined according to the ELISA procedures described previously [30]. The C1, C3 and C5 samples were simultaneously determined with graphite furnace atomic absorption spectroscopy (GFAAS).

### Preparation and immunofluorescent staining of tissue sections

After the treatment period, selected roots were quickly washed with deionized water and cut transversely with a razor blade into 3 cm-long segment in the root hair zone. The segments were immediately and completely embedded in OCT embedding compound and then frozen at -60°C. The sample segments were sectioned crosswise at 10 μm thick using a Microm HM525 cryostat from Microm Instruments, Inc. (San Macros, CA) and mounted on microscopic glass slides. Sections were air dried for 30 min at room temperature to prevent them from falling off the slides during antibody incubations. The tissue sections were prefixed in 5-mM ITCB-EDTA solution for 10 min at 4°C, then fixed in a FAA solution for another 10 min. After the FAA solution was discarded, the sections were washed gently 3 times with PBS.

The slides were incubated in PBS containing 1% BSA for 10 min at room temperature to block non-specific binding sites. After the BSA/PBS solution was washed off, mMb4F_3_B_6_D_9_A_1_ in PBS buffer was placed on the tissue followed by incubation for 1 h at room temperature. After removal of the primary antibody mMb4F_3_B_6_D_9_A_1_, the FITC-labeled anti-mouse IgG secondary antibody in PBS was added and then incubated for 1 h at room temperature. The optimum working dilutions for the primary antibody and FITC-labeled secondary antibody were 1:500 and 1:50, respectively. For the control test, the tissue sections were treated in the same manner as the treatment described above, except without mMb4F_3_B_6_D_9_A_1_. The stained sections were visualized on a Nikon C1 Plus LCSM microscope equipped with a 488-nm laser for excitation.

### Preparation and immunoelectronic staining of tissue sections

The selected roots were quickly rinsed with deionized water and cross-sectioned with a razor blade into 1 mm tissue blocks. The tissue sections were immediately fixed in 53.1 mM ITCB-EDTA solution at 4°C for 15 to 30 min followed by a mixture of 10% paraformaldehyde and 1% glutaraldehyde in 0.1 M PBS. They were entirely infiltrated with these solutions under brief vacuum. After fixation for approximately 8 h at room temperature, the samples were washed in 0.1 M PBS and dehydrated sequentially in a series of ethanol solutions (30%-100% in a 10% increment). The tissue sections were infiltrated and embedded in LR White resin. Ultrathin sections (70–80 nm) of the treated samples were obtained using a Leica EM UC6 Ultra-S microtome and then mounted onto nickel grids (50 mesh).

Immunoelectronic tissue staining was all performed at ambient temperature. Ultrathin tissue sections were treated sequentially with the following solutions (each 30 μL): 50 mM glycine in 0.1 M PBS buffer for 5 min; 1% BSA in PBSTG for 5 min; mMb4F_3_B_6_D_9_A_1_ in PBSGT (containing 1% BSA) for 60 min; rinse with PBS 3 times; 10 nm colloidal gold-labeled anti-mouse IgG secondary antibody in PBSTG for 45 min; rinse with ultrapure water 3 times; 1% aqueous uranyl acetate for 30 min; rinse with ultrapure water3 times. For immunoelectronic staining, the optimum working dilutions for the primary antibody and colloidal gold-labeled anti-mouse IgG secondary antibody were 1:1000 and 1:100, respectively. All the specimens were finally observed under a JEM-1230 transmission electron microscope with an energy-dispersive X-ray (EDX) analyzer.

### Conventional metal-S coprecipitation

The sample preparation for the conventional Cd^2+^-S coprecipitation method was performed as described previously [31]. Tissue segments were washed with deionized water and then fixed in the presence of 1% Na_2_S with 3% glutaraldehyde in 0.2 M phosphate buffer (pH 7). Post-fixation in 1% OsO_4_ was performed before dehydration with ethanol series (50, 60, 70, 80, 90, and 100%). After dehydration, samples were treated with propylene oxide and infiltrated in Spurr resin. The segments were then cut into ultrathin slides (70–80 nm). Prior to final observation under the JEM-1230 transmission electron microscope, the specimens were stained with 1% aqueous uranyl acetate for 15 min followed by 1% lead citrate for 15 min.

## Results

### Cadmium accumulation in wheat

After 28 days of exposure, toxicity symptoms such as quite noticeable browning, shortening and thickening of roots, were observed when the fortified Cd^2+^ content in the soil was greater than 50 μg g^-1^. The concentrations of Cd^2+^ in the wheat plants were determined with icELISA and confirmed with GFAAS. Cd^2+^ accumulation in the roots and leaves of the wheat plants cultivated in soil fortified with different concentrations of Cd^2+^ is shown in Table 1. Cd^2+^ concentrations in the roots were higher than those in the leaves. The translocation factor (TF) is the ratio of metal concentration in other plant parts to that in plant roots [32] and is an important parameter in studies of metal accumulation and translocation. All the TF values of Cd^2+^ from roots to leaves were <1, which indicate a strong capability to hold Cd^2+^ in root cells against transport to the aboveground parts of wheat plants.

### Fixation of Cd^2+^ in plant tissue

**Fig 1:**
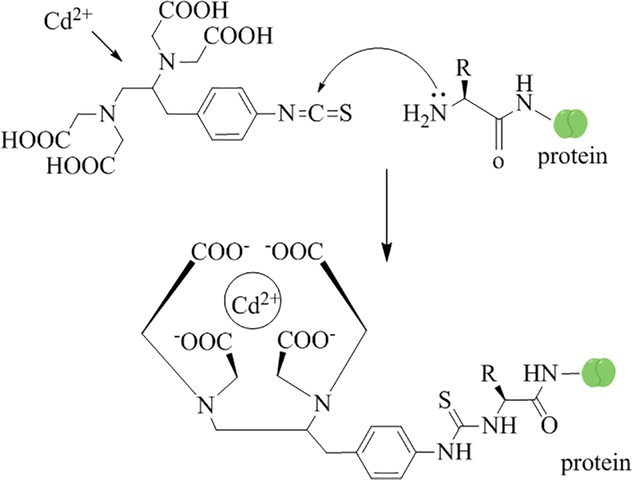
Proposed mechanism of immunohistochemical prefixation of Cd^2+^ in plant tissues.

The major obstacles to developing an IHC method for metallic ions are preserving tissue structure integrity as close to the living situation as possible [33] and avoiding loss and redistribution of ions. To overcome this challenge, ITCB-EDTA was used to chelate the mobile Cd^2+^ in the plant tissue and fix to proteins at the site via the isothiocyano functional group prior to fixation of frozen tissue samples or fixation for transmission electron microscopy. Fig 1 shows the proposed mechanism of IHC Cd^2+^ prefixation in plant tissues. ITCB-EDTA solution was applied to bind the mobile Cd^2+^ to the surrounding proteins, while mAb4F_3_B_6_D_9_A_1_ recognized the EDTA-Cd^2+^ portion of the ITCB-EDTA-Cd^2+^ complex. For immunofluorescent staining, the optimum working dilutions for the primary antibody (mAb4F_3_B_6_D_9_A_1_) and FITC-labeled secondary antibody were 1:500 and 1:50, respectively. For immunoelectronic staining, the optimum working dilutions for the primary antibody and colloidal gold-labeled anti-mouse IgG secondary antibody were 1:1000 and 1:100, respectively.

### Immunofluorescent staining for Cd^2+^

**Fig 2:**
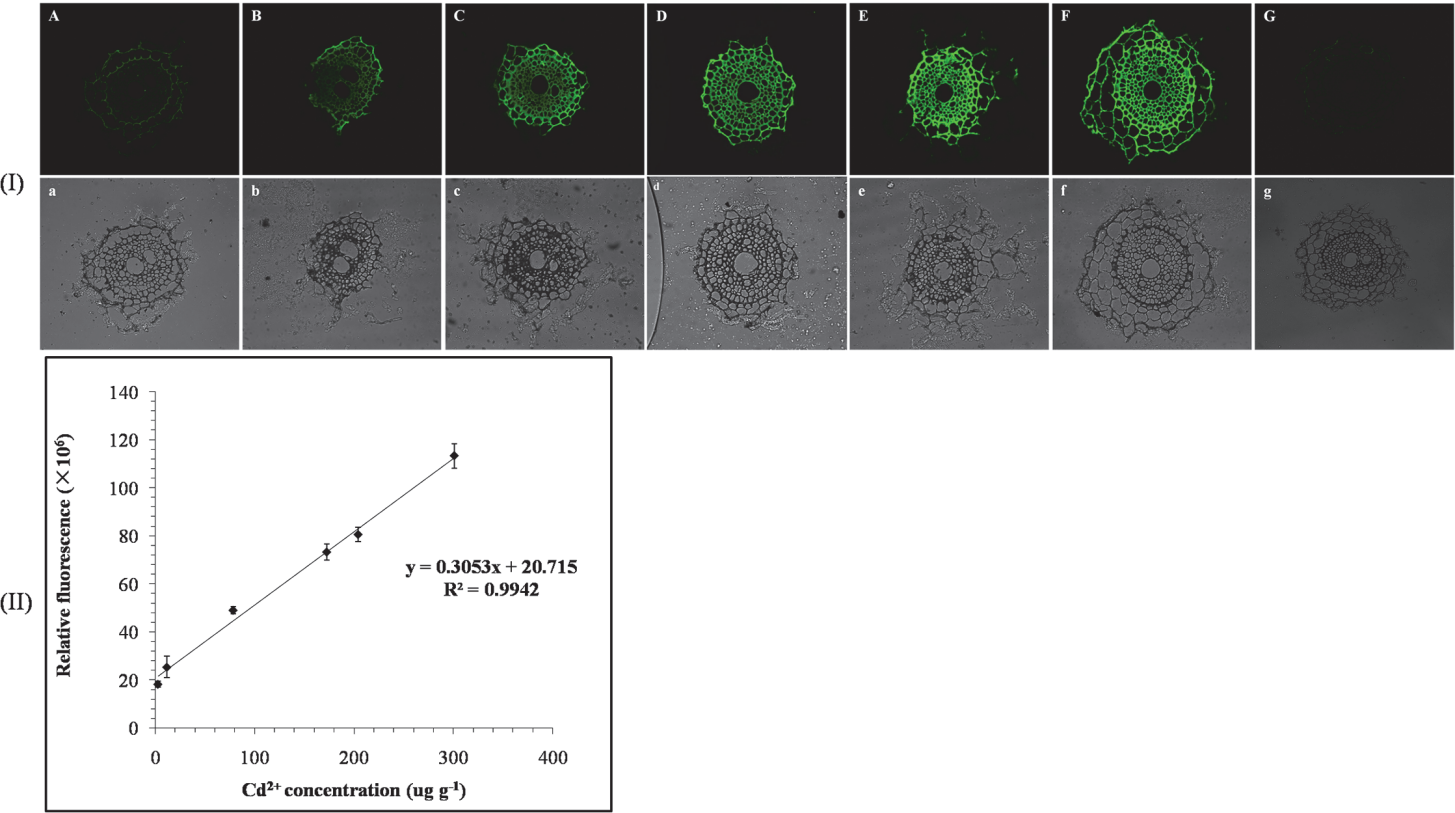
Quantitative immunohistochemical images (I) and quantitative relation (II). (I) Fluorescence microscopic images of Cd^**2+**^ distribution in the roots of wheat plants exposed to Cd^**2+**^ at 0, 1, 5, 25, 50 and 200 μg g^**-1**^ (A-F, respectively), image of no primary antibody control tests (G), and corresponding bright field images (a-g). Cd^**2+**^ was immunohistochemically localized with mAb4F_3_B_6_D_9_A_1_ and ITCB-EDTA. (II) The quantitative relation between the Cd^**2+**^ content and relative fluorescent value measured in A-F. The error bars represent standard derivations of triplicate measurements.

Fig 2I A-F show fluorescent microscopic images of Cd^2+^ distribution in wheat exposed to different Cd^2+^ doses (0, 1, 5, 25, 50 and 200 μg g^-1^) for 28 days, and Fig 2I G shows the image of no primary antibody control tests, while Fig 2I a-g show the corresponding bright field images. Except for the images of the Cd^2+^-negative tissue section (Fig 2I A) and control treatment (Fig 2I G), bright green fluorescence could be observed in all other images of Cd^2+^-treated wheat. There was a distinct enhancement of the root cross-sectional area and the green fluorescent signal intensity with increased concentrations of Cd^2+^ in the soil. For plants that grew in soil fortified with 1 and 5 μg g^-1^ Cd^2+^, bright green fluorescence could be observed mainly in the endodermis, and dots of fluorescence signal could be also observed in the stele cells (Fig 2I B and C). When the Cd^2+^ concentrations in the soil were 25 and 50 μg g^-1^, bright fluorescence could be observed in the stele (Fig 2I D and E). When the Cd^2+^ concentration was 200 μg g^-1^, extremely bright fluorescence was observed throughout the whole root (Fig 2I F). The results indicated that at low concentrations, Cd^2+^ principally precipitated within and outside the endodermis. When the Cd^2+^ concentration in the soil was higher than 25 μg g^-1^, excess Cd^2+^ passed through the endodermis and entered the stele. In unexposed plants and controls, a faint but not bright signal within the background range was still observed, probably as a result of autofluorescence produced by polyphenols and other compounds [14].

Leitenmaier and Küpper [18] reported that fluorescence microscopy cannot quantitatively detect target metal ions in cells because the dyes do not display high selectivity for the target metals, and the fluorescence response of the dye to metal concentrations is not linear. In this experiment, a standard curve relating the total FITC fluorescent value and the Cd^2+^concentration in the plant roots was developed (Fig 2II). The mean correlation coefficient of the standard curve was 0.9955, which demonstrated that the immunofluorescent method for Cd^2+^ was sensitive and specific enough for the quantitative imaging of Cd^2+^ in plant tissues. The fluorescent value was expressed as the product of the average fluorescent intensity and its area, which was obtained by Nikon EZ-C1 Free Viewer software.

### Energy-dispersive X-Ray confirmation of Cd^2+^ subcellular location detected with the immunoelectronic method

**Fig 3:**
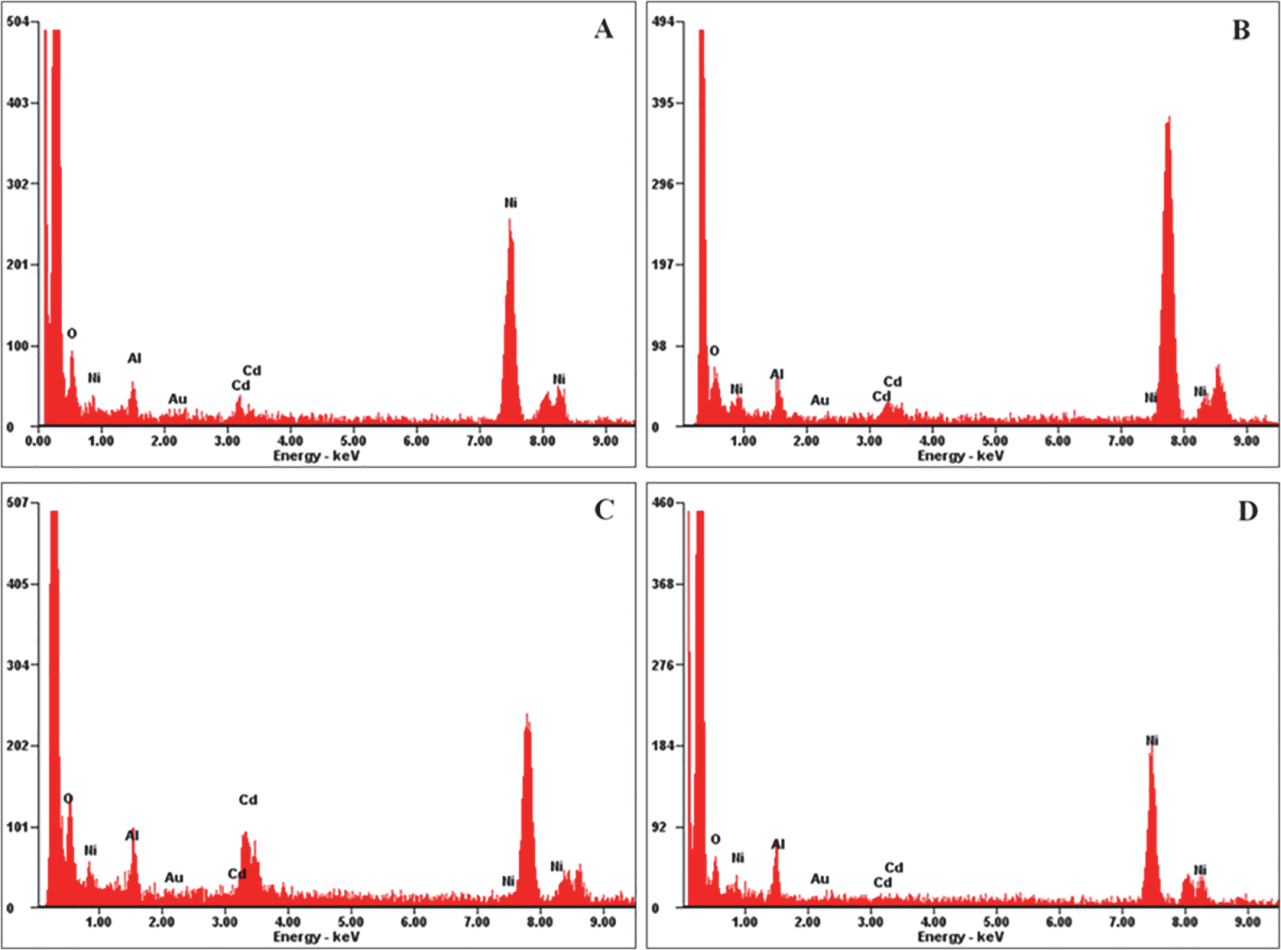
Energy-dispersive X-ray (EDX) analysis of the immunohistochemical method. EDX spectra of metals in xylem cells (A), endodermis cells (B), curdled macromolecular substances in the vacuole (C) and a region where there were no gold particles, indicating no Cd^**2+**^ deposition (D). The EDX spectra show the specific detection of Cd^**2+**^ in the different compartments of wheat plants grown in soil supplemented with 100 μg g^**-1**^ Cd^**2+**^. The Al and Ni peaks were from the Al holder and Ni grid, respectively.

In addition to the immunofluorescent method, a new immunoelectronic staining method was developed and confirmed with EDX analysis. To verify whether gold particles can represent the location of Cd^2+^ in the plant cell, gold particles from the colloidal gold-labeled anti-mouse IgG secondary antibody were analyzed randomly on an EDX analyzer. Fig 3A–3C shows the EDX spectra of the gold particles. The particles showed the specific Cd^2+^ location in the xylem and endodermis cells and curdled macromolecular substances of wheat plants exposed to 100 μg g^-1^ Cd^2+^. Fig 3D shows the spectrum of a blank region without gold particles. The Cd peak was present in each gold particle EDX spectrum (Fig 3A–3C) but was not present in the spectrum of the blank region. The peaks labeled Al and Ni are the Al holder and Ni grid, respectively. The EDX spectra of the gold particles and blank region proved the reliability of the established immunoelectronic microscopic method.

### Subcellular localization of Cd^2+^ with the conventional Na_2_S precipitation method

**Fig 4:**
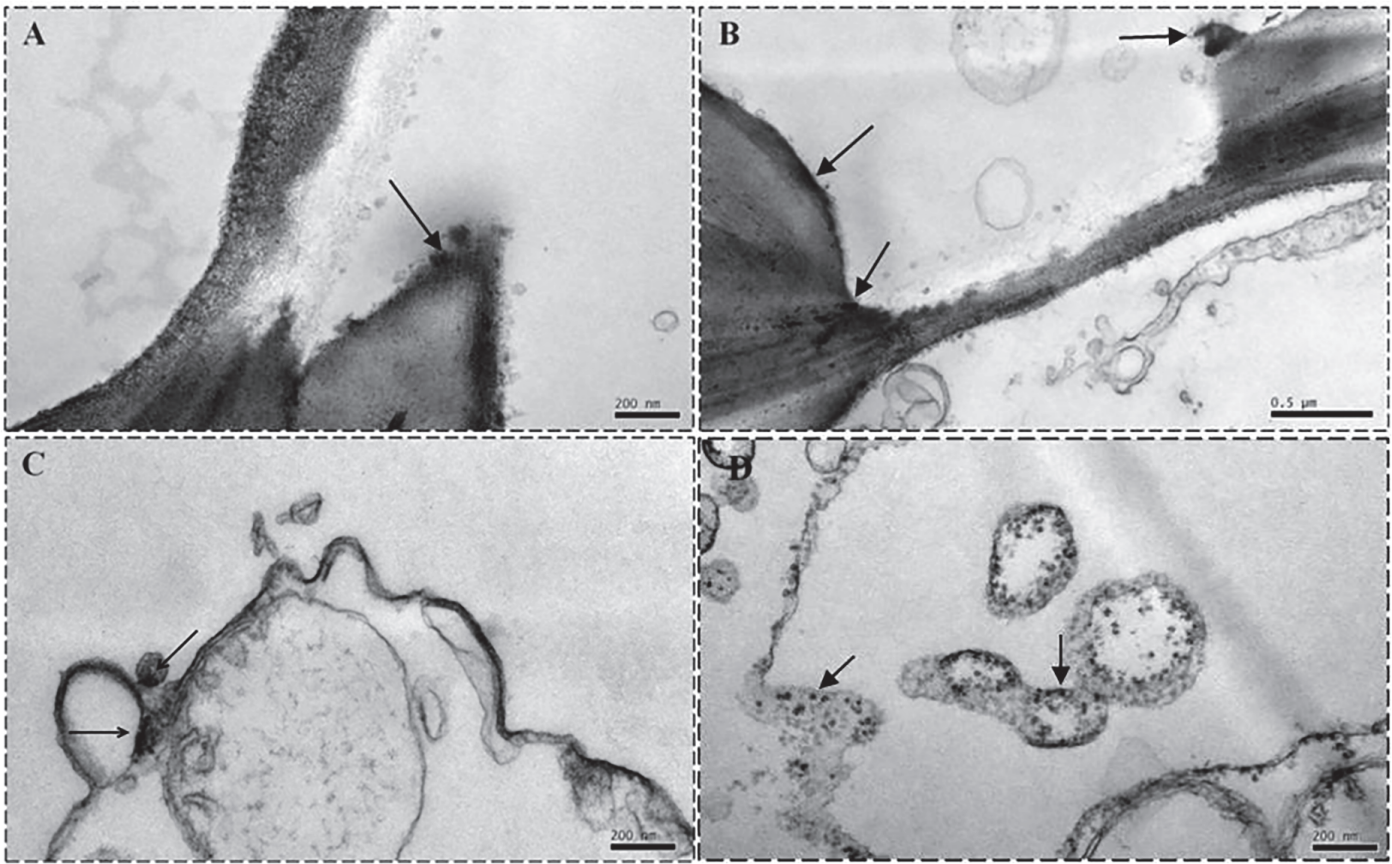
Cd^2+^ distribution in the stele cells of wheat roots with metal-S coprecipitation method. Electron microscope images of Cd^**2+**^ distribution in the cell walls (A and B) and plasma membrane (C and D) of root stele cells of wheat plants grown in soil fortified with 0 (A and C) and 100 μg g^**-1**^ Cd^**2+**^ (B and D). Cd^**2+**^ was localized with the conventional metal-S coprecipitation histochemical method. Arrowheads indicate Cd^**2+**^ deposits.

**Fig 5:**
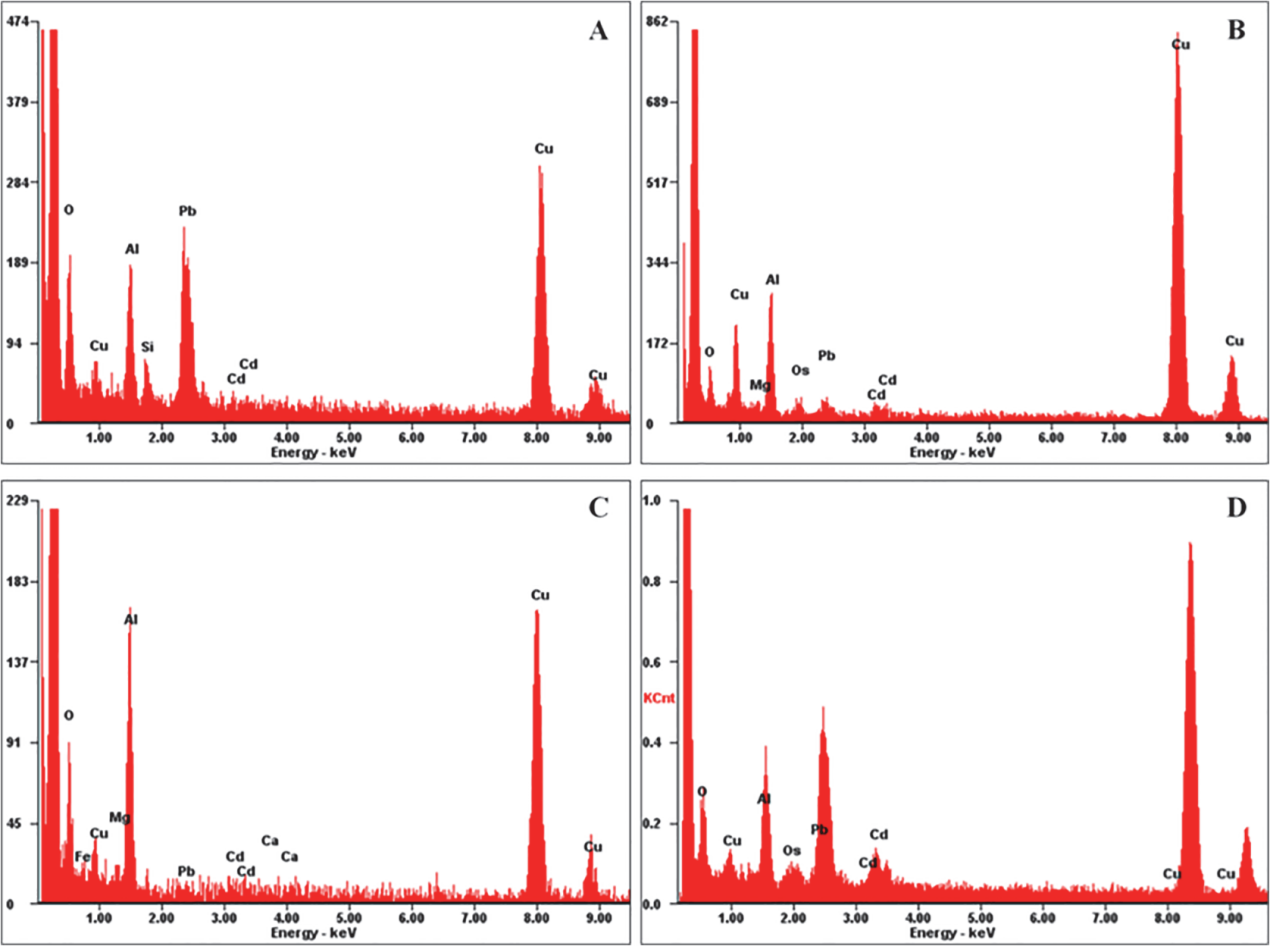
EDX analysis of the metal-S coprecipitation method. EDX spectra of Cd^**2+**^ deposits near the cell wall (A and B) and curdled macromolecular substances (C and D) of wheat plants grown in soil fortified with 0 (A and C) and 100 μg g^**-1**^ Cd^**2+**^ (B and D). Cd^**2+**^ was localized with the conventional metal-S coprecipitation histochemical method. Note: The EDX spectra suggest that the illustration of Cd^**2+**^ deposition detected with the traditional histochemical method as shown in Fig 4 may be interfered by other metal ions such as Mg^**2+**^.

The conventional histochemical method of Na_2_S precipitation for heavy metal ions was also implemented for comparison with the IHC method developed in the present study. Fig 4 shows the Cd^2+^ distribution in the stele of Cd^2+^-negative (Fig 4A and 4C) and Cd^2+^-treated (Fig 4B and 4D) wheat plants. The dark deposits in the wheat plants were analyzed randomly on an EDX analyzer, and the corresponding spectra are displayed in Fig 5. The deposits in the Cd^2+^-negative wheat sample (Fig 4A and 4C) suggest that Na_2_S precipitation method could produce false positive, and the corresponding spectra (Fig 5A and 5C) proved the standpoint, which had Cu and Mg peak, and no obvious Cd peak. The spectrum of the Cd^2+^-treated samples had an obvious Cd peak, Mg peak and Cu peak (Fig 5B), which also suggest that it is lack of specificity for Cd^2+^ using the Na_2_S precipitation method for heavy metal ion detection, an inherent disadvantage of the method [12]. Note: the peaks labeled Al, Os, Pb and Cu indicate the Al holder, osmium tetroxide fixative, lead citrate staining solution and Cu grid, respectively.

### Adsorption dynamics of Cd^2+^

**Fig 6:**
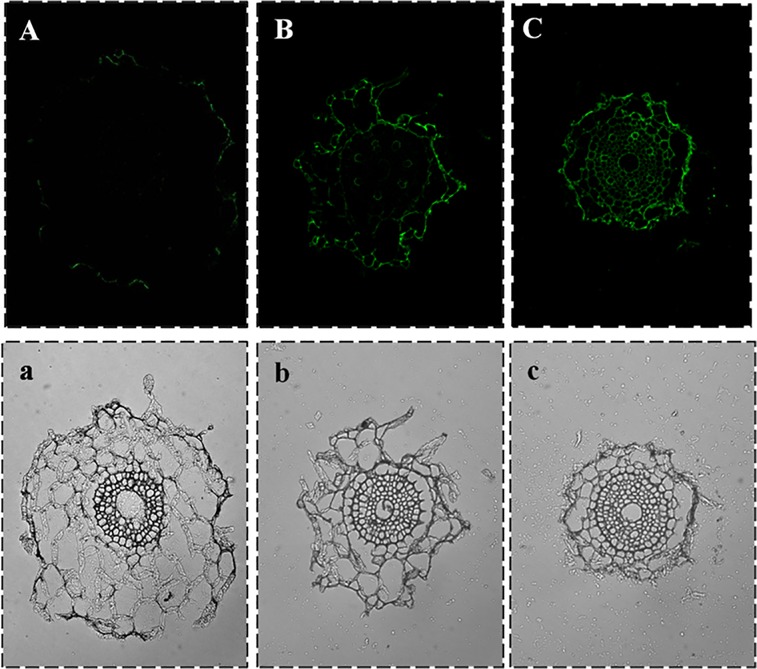
Dynamic distribution of Cd^2+^ in wheat roots. Fluorescence microscope images of the cellular distribution of Cd^**2+**^ in wheat roots 2, 4, and 15 days after germination (A, B and C, respectively) and the corresponding bright field images (a, b and c, respectively). Cd^**2+**^ was localized with the IHC method using mAb4F_3_B_6_D_9_A_1_ and ITCB-EDTA.

The immunofluorescent method developed in the present study was used to study the dynamic process of Cd^2+^ absorption in wheat roots exposed to 50 ug g^-1^Cd^2+^ (Fig 6). Samples were collected 2, 4 and 15 days after germination. The epidermis cells were the first binding sites for Cd^2+^. Bright green fluorescence could be observed only 2 days after seed germination (Fig 6A). As the exposure time increased, Cd^2+^ was observed in the xylem and then phloem; the xylem accumulated more Cd^2+^ than the primary phloem, as indicated by the fluorescence intensity (Fig 6B and 6C).

### Subcellular localization of Cd^2+^

**Fig 7:**
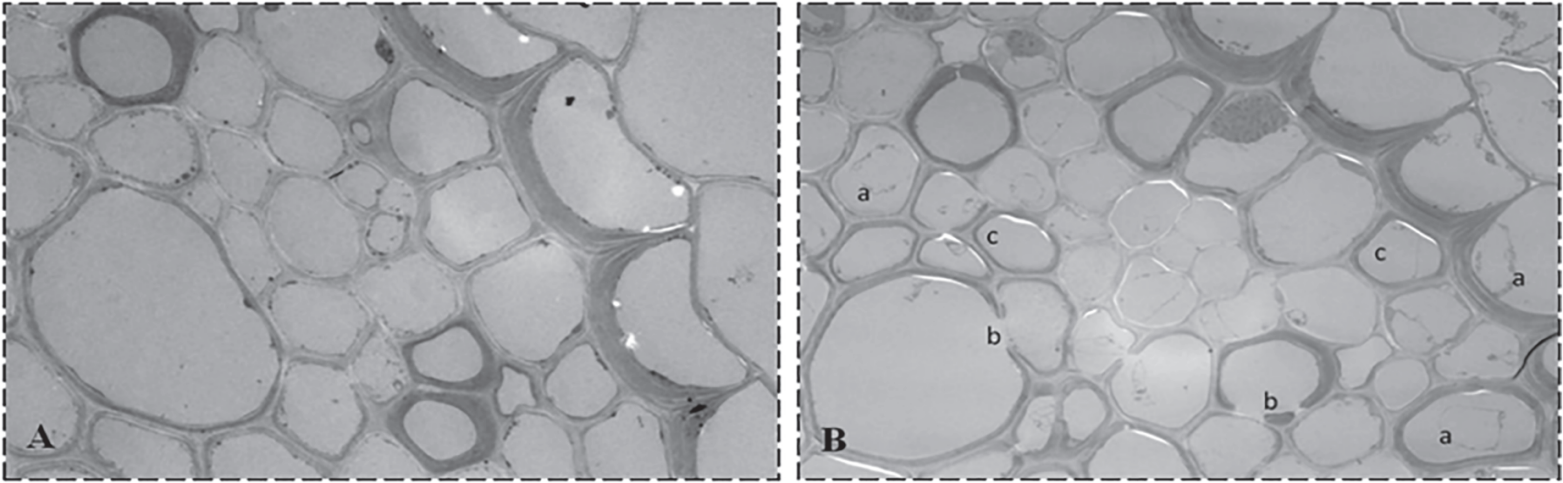
Immunoelectronic microscope images of wheat root transverse sections. (A) and (B) are a Cd^**2+**^-negative plant and one exposed to 100 μg g^**-1**^ Cd^**2+**^, respectively. Note: curdled macromolecular substances in the vacuole (a), cell wall fracture (b), and lignification (c) are shown in (B).

Fig 7 shows immunoelectronic microscopic images of wheat root transverse sections. Compared to the unexposed controls (Fig 7A), the cells of the Cd^2+^-treated wheat plants were smaller and more closely arranged, were more seriously lignified, and were structurally impaired, such as cell wall fracture [34] and coagulation of macromolecular substances in the vacuole.

**Fig 8:**
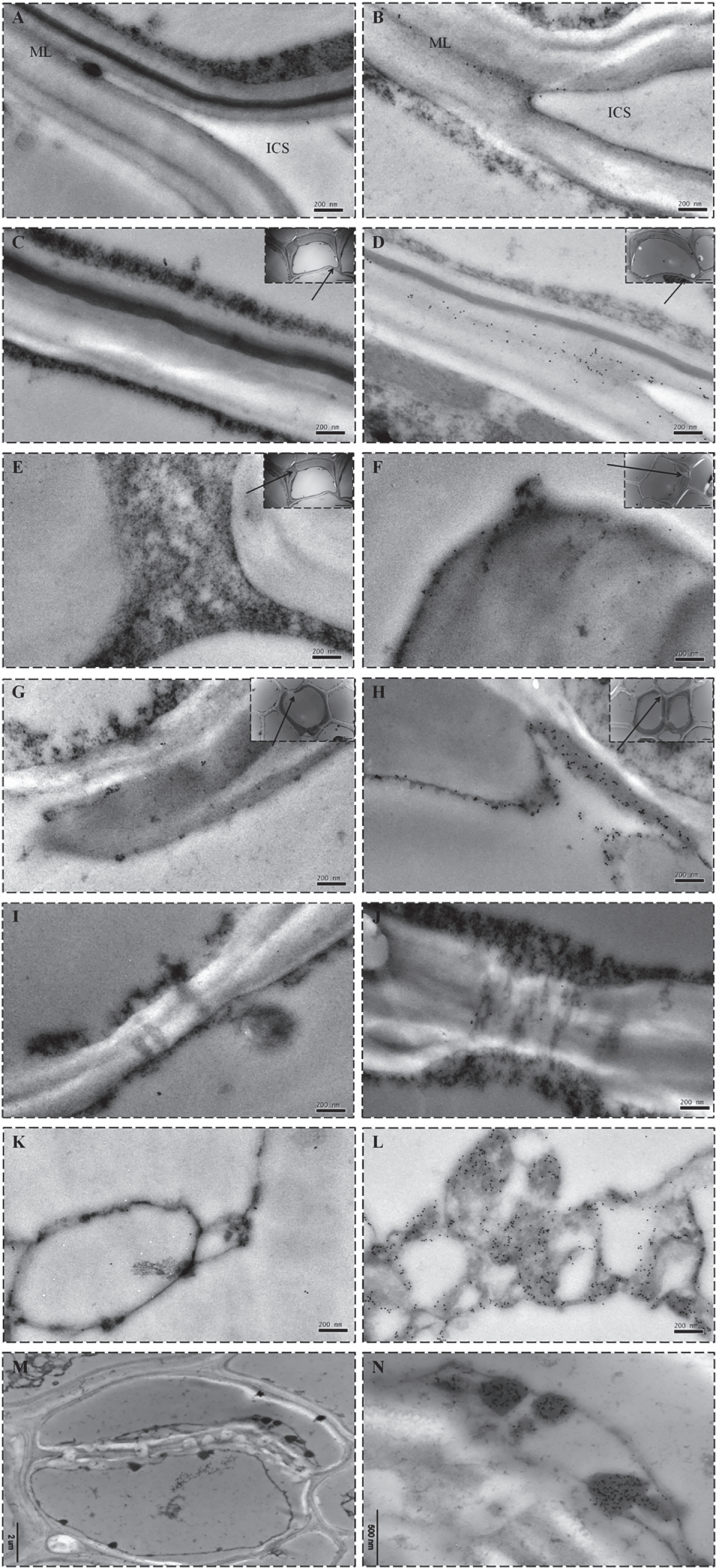
Cd^2+^ subcellular distribution in different cellular compartments of wheat roots. Comparison of immunoelectronic microscope images of Cd^**2+**^ distribution in the cell walls of the cortex (A, B), outer tangential wall (C, D) and inner tangential wall (E, F) of endodermal cells, the cell membrane of xylem cells (G and H), plasmodesmata (PD) of phloem cells (I and J), and curdled macromolecular substances in the vacuole (K and L) of root vascular cells of wheat roots from a Cd^**2+**^-negative plant (A, C, E, G, I and K) and a plant treated with 100 μg g^**-1**^ Cd^**2+**^ (B, D, F, H, J and L). Cd^**2+**^ was detected with the IHC method. Colloidal gold particles were only observed in the cortical tissue of the middle lamella (ML) and the outer surface of the cell wall in the intercellular space (ICS) (B), the outer tangential endodermis wall (D), the membrane along the inner tangential cell wall (F), the plasma membrane near the xylem cell wall (H), PD (J) and curdled proteinaceous material (L) in the vacuole and membrane-bound organelles (M and N) of phloem cells in Cd^**2+**^-positive plants. ICS is the intercellular space, and ML is the middle lamella. The insets display the magnified regions, and the arrowheads indicate Cd^**2+**^ depositions.

Fig 8 shows the location of Cd^2+^ deposition in the cortex, endodermis and stele cells of control and Cd^2+^-treated wheat roots. In the Cd^2+^-treated plants (Fig 8B), distinct gold particles were observed in the cortical tissue of the middle lamella and the outer surface of the cell wall in the intercellular space bordered by three cortical cells, whereas few particles were observed in the control plants (Fig 8A).On the outer and inner tangential endodermal cell wall of Cd^2+^-treated plants (Fig 8C and 8E), there were no distinct colloidal gold particles. Fig 8D shows that the non-thickened outer tangential endodermal wall of the Cd^2+^-treated plant was one of the main Cd^2+^ deposition locations. However, only a few gold particles appeared on the membrane along the inner tangential cell wall and none on the inner tangential wall (Fig 8F). Fig 8H shows Cd^2+^-induced cell wall fracture and Cd^2+^ deposition in the cytoplasm near the cell wall, which were not observed in Cd^2+^ control plants (Fig 8G). To date, there is no direct evidence that Cd^2+^ can be transported through the symplast pathway. Gold particles were observed in plasmodesmata (PD) of Cd^2+^-treated plants (Fig 8J) but not in control plants (Fig 8I). There was notable difference in gold particle density on the curdled macromolecular substances in the vacuole between the control plant (Fig 8K) and the Cd^2+^-treated plant (Fig 8K). In addition, more membrane-bound organelles were found in some root phloem cells of Cd^2+^-positive plants compared to the control (Fig 8M), and numerous gold particles were observed (Fig 8N).

## Discussion

Plant roots absorb metals that are in a highly soluble and mobile form. Once inside plant cells, metal ions can bind to various molecules, including organic acids and bases, polysaccharides and proteins in plant cells [5]. Autometallography [2,3,10,11] and the conventional histochemical method of Na_2_S precipitation of heavy metal ions [12,20,31] are based on the reaction of free metal ions such as Cd^2+^ with Na_2_S to form metallic sulfur deposits (e.g., CdS), which can mark the location of free heavy metal ions. Similarly, the metal-specific fluorescent dyes [18,19] used in fluorescence microscopy based on chelation of metal ions by fluorescent dyes and also show the location of free metal ions. PIXE analysis [16,17] and autoradiography can visualize all forms of metals including free and immobile metal ions.

The IHC method developed in the present study can show the location of free Cd^2+^. The major obstacle of an IHC method for small molecules is how to fix the target molecule to tissue protein while preserving the protein’s antigenicity. For example, the principle of fixation of the plant hormones indole-3-acetic acid, abscisic acid and gibberellic acid is that the carboxyl group of the target molecule is activated with 1-(3-demethylaminoprapyl)-3-ethyl carbodiimide and then coupled with the amino group of tissue protein [26–29]. Unlike hormones, metal ions have no active group to be coupled to the tissue protein.

The bifunctional reagent ITCB-EDTA was used to immobilize Cd^2+^ in plant tissues prior to fixation with FAA solution or a mixture of 10% paraformaldehyde and 1% glutaraldehyde. The mechanism of IHC Cd^2+^ prefixation in plant tissues is that ITCB-EDTA binds free Cd^2+^ to form an ITCB-EDTA-Cd^2+^ complex that is then fixed to proteins at the site via the complex’s isothiocyano functional group (Fig 1). The ITCB-EDTA-Cd^2+^ complex is recognized by mAb4F_3_B_6_D_9_A_1_, which is specific and sensitive to Cd^2+^-EDTA. This cross-reactivity of this primary mAb with non-target metal-EDTA complexes was below 1%, except for Mn^2+^ (2.9%) and Hg^2+^ (1.6%) [30], which assured specific IHC localization of Cd^2+^.

The effective fixation of Cd^2+^ made the development of an immunofluorescent staining method possible. The IHC method was used to examine the relationship between the total FITC fluorescence intensity and the Cd^2+^concentration in the plant roots as well as the Cd^2+^ distribution. The results demonstrated that the immunofluorescent method was suitable for specific localization and quantification of Cd^2+^in plant tissues (Fig 2II). Fig 2I shows the Cd^2+^ distribution in wheat roots exposed to different Cd^2+^ doses for 28days. The results suggest that (1) Cd^2+^ could induce a distinct enhancement of the root cross-sectional area, cell number and cell layers to hold more Cd^2+^ in the roots, and (2) with increased concentration of Cd^2+^ in the soil, Cd^2+^ precipitation was extended from the exodermis (Fig 2I B) to the whole root (Fig 2I F). It can be concluded that the cortical apoplast is the primary site of Cd^2+^ deposition in the root. But there is still another possibility that with the increase of exposure time, Cd^2+^ could pass through the endodermis and enter the vascular cells, even when the Cd^2+^ concentrations was 1 μg g^-1^. Because of their low spatial resolution and nonspecificity, the Cd^2+^ histochemical methods described in the literature cannot completely identify the subcellular localization of heavy metals in plants. The immunoelectronic microscopic method developed in the present study has overcome these problems and was used to study the subcellular location of Cd^2+^ in the root cells (Fig 8).

Studies have shown that the main site of Cd^2+^ accumulation in roots is the apoplast [20,35,36]. In the present experiment, distinct gold particles were observed in the middle lamella and the cell walls of the epidermal, cortex and endodermal cells. Similarly, Vázquez *et al*. [37] showed that the main locations of Cd accumulation in *Thlaspi caerulescens* roots were cell walls, and the specific localization of Cd in the middle lamella suggests that Cd is principally bound to ion exchange sites on pectic residues. Khan *et al*. [38] found Cd only in the cell walls of the sieve elements and in the middle lamella separating the endodermis in roots of *Zea mays*. In addition, proteinaceous materials clotting appeared in the vacuole of some cortical cells (Fig 7B), and numerous Cd^2+^ ions aggregated on them like Fig 8L.

The phenomenon of horseshoe-shaped thickening of the endodermal cell walls in the plants is generally accepted, but so far there is no definitive evidence to prove the function and effect of thickened cell walls. Fig 8D shows abundant gold particles on the non-thickened outer tangential wall of the endodermis but not on the inner tangential wall, and only a few appeared on the membrane (Fig 8F). The results demonstrates that the horseshoe-shaped thickening of the inner tangential endodermis cell walls provides an effective barrier and effectively hampers Cd^2+^entry into the stele by apoplastic transport, so Cd^2+^ was forced into symplasts. This observation also can explain why gold particles cannot be observed on the apoplast of the stele (Fig 8H).

The mechanism of Cd^2+^ transportation in the stele is still not clear. In this experiment, some direct visual evidence was observed. Fig 8H and 8J indicate that the symplast pathway through the plasma membrane of fractured xylem cells (Fig 8H) and PDs of phloem cells (Fig 8J) is an important route of Cd^2+^ transport in wheat plants. The PD is a unique channel structure that spans plasma membranes and cell walls between adjacent plant cells, creating the symplast, and is involved in the exchange of a wide range of molecules, including ions, water, proteins and nucleic acids [25]. In addition, abundant gold particles were observed on curdled macromolecular substances in the vacuole (Fig 8L) and membrane organelles (Fig 8N) of phloem cells. Cd^2+^ accumulation in the vacuole and membranes organelles could also be a retention mechanism for plants protecting the plasma membrane and keeping toxic ion concentration low in the cytoplasm [34,39], to further inhibit the Cd^2+^ transport into the xylem and eventually the aboveground parts of the plant. This result explains why the Cd^2+^ concentrations in roots were higher than those in leaves (Table 1).

## Conclusions

This study developed an IHC localization method for Cd^2+^ in plant tissue. This method employed ITCB-EDTA to chelate free Cd^2+^ in the tissue prior to conventional tissue fixation protocols to convert Cd^2+^ to Cd^2+^-EDTA, which can be recognized and bound by mAb4F_3_B_6_D_9_A_1_. The IHC localization results show distinct differences in the fluorescent signal intensity between Cd^2+^-positive and Cd^2+^-negative samples, and colloidal gold particles were only observed in the Cd^2+^-positive samples. Compared to the conventional metal-S coprecipitation method, this new IHC method is quantitative, more specific to Cd^2+^ and has less background interference. In addition, we observed the dynamic distribution of Cd^2+^ in wheat roots using the immunofluorescent method and Cd^2+^ subcellular distribution in different cellular compartments of wheat roots using the immunoelectronic method. The localization results also illustrate the Cd^2+^ translocation pathway.

## References

[1] Huff J, Lunn RM, Waalkes MP, Tomatis L, Infante PF. Cadmium-induced cancers in animals and in humans. International Journal of Occupational and Environmental Health. 2007; 13: 202–212. 17718178 10.1179/oeh.2007.13.2.202 PMC3399253

[2] Garg N, Aggarwal N. Effects of interactions between cadmium and lead on growth, nitrogen fixation, phytochelatin, and glutathione production in mycorrhizal *Cajanuscajan* (L.) Millsp. Plant Growth Regul, 2011; 30: 286–300.

[3] Vassilev A. Physiological and agroecological aspects of cadmium interactions with barley plants: an overview. Journal of Central European Agriculture. 2002; 4: 66–76.

[4] Puschenreiter M, Horak O, Friesl W, Hartl W. Low-cost agricultural measures to reduce heavy metal transfer into the food chain-a review. Plant Soil Environ. 2005; 51: 1–11.

[5] Zeng F, Zhou W, Qiu B, Shafaqat A, Wu F, Zhang G. Subcellular distribution and chemical forms of chromium in rice plants suffering from different levels of chromium toxicity. 2011; J. Plant Nutr. Soil Sci. 174: 249–256.

[6] Vazqueza S, Fernandez-Pascual M, Sanchez-Pardo B, Carpena RO, Zornoza P. Subcellular compartmentalisation of cadmium in white lupins determined by energy-dispersive X-ray microanalysis. J. Plant Physiol. 2007; 164: 1235–1238. 17434645 10.1016/j.jplph.2006.11.011

[7] Wang Z, Nan Z, Wang S, Zhao Z. Accumulation and distribution of cadmium and lead in wheat (*Triticum aestivum* L.) grown in contaminated soils from the oasis, north-west China. J. Sci. Food Agric. 2011; 91: 377–384. 10.1002/jsfa.4196 21086461

[8] Van Belleghem F, Cuypers A, Semane B, Smeets K, Vangronsveld J, d'Haen J, et al Subcellular localization of cadmium in roots and leaves of *Arabidopsis thaliana* . New Phytol. 2007; 173: 495–508. 17244044 10.1111/j.1469-8137.2006.01940.x

[9] Dinh NT, Vu DT, Mulligan D, Nguyen AV. Accumulation and distribution of zinc in the leaves and roots of the hyperaccumulator *Noccaea caerulescens* . Environ. Exp. Bot. 2015; 110: 85–95.

[10] Heumann H. Ultrastructural localization of zinc in zinc-tolerant *Armeria maritima* ssp. *halleri* by autometallography. J.Plant Physiol. 2002; 159: 191–203.

[11] Vollenweider P, Cosio C, Unthardt-Goerg MSG, Keller C. Localization and effects of cadmium in leaves of a cadmium-tolerant willow (*Salix viminalis* L.) Part II microlocalization and cellular effects of cadmium. Environ. Exp. Bot. 2006; 58: 25–40.

[12] Wójcik M, Vangronsveld J, Haenc JD, Tukiendorf A. Cadmium tolerance in *Thlaspi caerulescens*: II. Localization of cadmium in *Thlaspi caerulescens* . Environ. Exp. Bot. 2005; 53: 163–171.

[13] Cosio C, DeSantis L, Frey B, Diallo S, Keller C. Distribution of cadmium in leaves of *Thlaspi caerulescens* . J. Exp. Bot. 2005; 56: 765–775. 15642714 10.1093/jxb/eri062

[14] Ma JF, Ueno D, Zhao F, McGrath SP. Subcellular localisation of Cd and Zn in the leaves of a Cd-hyperaccumulating ecotype of *Thlaspi caerulescens* . Planta. 2005; 220: 731–736. 15517354 10.1007/s00425-004-1392-5

[15] McRae R, Bagchi P, Sumalekshmy S, Fahrni CJ. In situ imaging of metal in cells and tissues. *Chem*. *Rev*. 2009; 109: 4780–4827. 10.1021/cr900223a 19772288 PMC3812694

[16] Collins RN, Bakkaus E, Carrire M, Khodja H, Olivier P, Jean-louis M, et al Uptake, localization, and speciation of cobalt in *Triticu maestivum* L. (Wheat) and *Lycopersicon esculentum* M. (Tomato). Environ. Sci. Technol. 2010; 44: 2904–2910. 10.1021/es903485h 20345097

[17] Vogel-Mikuš K, Regvar M, Mesjasz-Przybyłowicz J, Przybyłowicz WJ, Simčič J, Pelicon P, et al Spatial distribution of cadmium in leaves of metal hyperaccumulating *Thlaspi praecox* using micro-PIXE. New Phytol. 2008; 179: 712–721. 10.1111/j.1469-8137.2008.02519.x 18554265

[18] Leitenmaier B, Küpper H. Cadmium uptake and sequestration kinetics in individual leaf cell protoplasts of the Cd/Zn hyperaccumulator *Thlaspi caerulescenspce* . Plant Cell Environ. 2011; 34: 208–219. 10.1111/j.1365-3040.2010.02236.x 20880204

[19] Lu L, Tian S, Yang X, Wang X, Brown P, Li T, et al Enhanced root-to-shoot translocation of cadmium in the hyperaccumulating ecotype of *Sedum alfredii* . J. Exp. Bot. 2008; 59: 3203–3213. 10.1093/jxb/ern174 18603654 PMC2504354

[20] Zhou Y, Huang S, Yu S, Gu J, Zhao J, Han Y, et al The physiological response and sub-cellular localization of lead and cadmium in *Iris pseudacorus* L. Ecotoxicology. 2010; 19: 69–76. 10.1007/s10646-009-0389-z 19629681

[21] Griesen D, Su D, Asard ABH. Localization of an ascorbate-reducible cytochrome b561 in the plant tonoplast. Plant Physiol. 2004; 134: 726–734. 14730083 10.1104/pp.103.032359 PMC344548

[22] Hao HQ, Chen T, Fan LS, Li RL, Wang XH. 2, 6-dichlorobenzonitrile causes multiple effects on pollen tube growth beyond altering cellulose synthesis in *Pinusbungeana Zucc* . Plos One. 2013; 8: 1–10. 10.1371/journal.pone.0076660 PMC3795706 24146903

[23] Astruc T, Marinova P, Labas R, Gatellier P, Santé-Lhoutellier V. Detection and localization of oxidized proteins in muscle cells by fluorescence microscopy. J. Agric. Food Chem. 2007; 5: 9554–9558. 10.1021/jf0717586 17941692

[24] Bauer M, Dietrich C, Nowak K, Sierralta WD, Papenbrock J. Intracellular localization of Arabidopsis sulfurtransferases. Plant Physiol. 2004; 135: 916–926. 15181206 10.1104/pp.104.040121 PMC514126

[25] Ueki S, Citovsky V. Identification of an interactor of cadmium ion-induced glycine-rich protein involved in regulation of callose levels in plant vasculature. Proc. Natl. Acad. Sci. USA. 2005; 102: 12089–12094. 16103368 10.1073/pnas.0505927102 PMC1189354

[26] Hou Z, Huang W. Immunohistochemical localization of IAA and ABP1 in strawberry shoot apexes during floral induction. Planta. 2005; 222: 678–687. 16001261 10.1007/s00425-005-0014-1

[27] Zhang M, Zheng X, Song S, Zeng Q, Hou L, Li D, et al Spatiotemporal manipulation of auxin biosynthesis in cotton ovule epidermal cells enhances fiber yield and quality. Nat. Biotechnol. 2011; 29: 453–458. 10.1038/nbt.1843 21478877

[28] Xu R, Niimi Y, Kojima K. Exogenous GA_3_ overcomes bud deterioration in tulip (*Tulipagesneriana* L.) bulbs during dry storage by promoting endogenous IAA activity in the internodes. Plant Growth Regul. 2007; 52: 1–8.

[29] Peng Y, Zou C, Wang D, Gong H, Xu Z, Bai S. Preferential localization of abscisic acid in primordial and nursing cells of reproductive organs of Arabidopsis and cucumber. New Phytol. 2006; 170: 459–466. 16626468 10.1111/j.1469-8137.2006.01683.x

[30] Gao W, Nan T, Tan G, Zhao H, Wang B, Li QX, et al Development of a sensitive monoclonal antibody-based enzyme-linked immunosorbent assay for the analysis of cadmium ions in water, soil and rape samples. Food and Agricultural Immunology. 2012; 23: 27–39.

[31] Solís-Domínguez FA, González-Chávez MC, Carrillo-González R, Rodríguez-Vázquez R. Accumulation and localization of cadmium in Echinochloa polystachya grown within a hydroponic system. J. Hazard Mater. 2007; 141: 630–636. 16920257 10.1016/j.jhazmat.2006.07.014

[32] Bose S, Bhattacharyya AK. Heavy metal accumulation in wheat plant grown in soil amended with industrial sludge. Chemosphere. 2008; 70: 1264–1272. 17825356 10.1016/j.chemosphere.2007.07.062

[33] Agar NYR, Yang HW, Carroll RS, Black PM, Agar JN. Matrix solution fixation histology-compatible tissue preparation for MALDI mass spectrometry imaging. Anal. Chem. 2007; 79: 7416–7423. 17822313 10.1021/ac071460e

[34] Sridhar BBM, Diehl SV, Han FX, Monts DL, Su Y. Anatomical changes due to uptake and accumulation of Zn and Cd in Indian mustard (*Brassica juncea*). Environ. Exp. Bot. 2005; 54: 131–141.

[35] Wójcik M, Tukiendorf A. Cadmium uptake, localization and detoxification in *Zea mays* . Biologia Plantarum. 2005; 49: 237–245.

[36] Zhao Y, Wu J, Shang D, Ning J, Zhai Y, Sheng X, et al Subcellular distribution and chemical forms of cadmium in the edible seaweed, *Porphyra yezoensis* . Food Chemistry. 2015; 168: 48–54. 10.1016/j.foodchem.2014.07.054 25172682

[37] Vázquez MD, Barceló J, Poschenrieder C, Mádico J, Hatton P, Baker AJM, et al Localization of zinc and cadmium in *Thlaspi caerulescens* (Brassicaceae), a metallophyte that can accumulate both metals. J. Plant Physiol. 1992; 140: 350–355.

[38] Khan DH, Duchett JG, Frankland B, Kirkham JB. An X-ray microanalytical study of the distribution of cadmium in roots of *Zea mays* L. J. Plant Physiol. 1984; 115: 19–28. 10.1016/S0176-1617(84)80047-4 23196083

[39] Han YL, Yuan HY, Huang SZ, Guo Z, Xia B, Gu J. Cadmium tolerance and accumulation by two species of *Iris* . Ecotoxicology. 2007; 16: 557–563. 17701346 10.1007/s10646-007-0162-0

